# Child-parent interactions in American and Turkish families: Examining measurement invariance analysis of child-parent relationship scale

**DOI:** 10.1371/journal.pone.0230831

**Published:** 2020-04-03

**Authors:** Elsa Lucia Escalante-Barrios, Sonia Mariel Suarez-Enciso, Helen Raikes, Dawn Davis, Aileen Garcia, Mubeccel Gonen, Mefharet Veziroglu-Celik, Ramle Gul Hazar

**Affiliations:** 1 Department of Education, Asociación de Investigación en Métodos Mixtos, ALIMM, Universidad del Norte, Atlántico, Colombia; 2 Department of Educational Psychology, Asociación de Investigación en Métodos Mixtos, ALIMM, University of Nebraska-Lincoln, Lincoln, Nebraska, United States of America; 3 Department of Child, Youth, and Family Studies, University of Nebraska-Lincoln, Lincoln, Nebraska, United States of America; 4 Department of Counseling and Human Development, South Dakota State University, Brookings, South Dakota, United States of America; 5 Department of Early Childhood Education, Hacettepe University, Ankara, Turkey; 6 Department of Early Childhood Education, Istanbul Medipol University, Istanbul, Turkey; 7 Early Childhood Education Department, Hacettepe University, Ankara, Turkey; Chinese Academy of Medical Sciences and Peking Union Medical College, CHINA

## Abstract

The parent-child relationship is a cornerstone of early childhood development and one-way early childhood programs can have a positive influence on early development is to adopt programmatic features to enhance this relationship. Research supports these conclusions in both U.S. and cross-cultural contexts, even though assumptions about parenting and the parent-child relationship may differ across cultures. However, for true understanding of cultural differences, it is important to have comparable measures across cultures. The purpose of the study is to assess measurement invariance of the two constructs of the Child-parent Relationship Scale using data gathered in programs serving low-income preschool children in the U.S.(*n* = 4,450) and Turkey (*n* = 592) from 2014 to 2015. Using Single-group Confirmatory Factor Analysis, the original factor structures of the Turkish and the English versions were tested. Besides, Multigroup Confirmatory Factor Analysis provided evidence for configural, metric, scalar invariance, strict factorial invariance or error variance invariance and construct level invariance across the two versions. Only configural invariance was established, which showed an agreement for the existence of an underlying theoretical construct for each subscale (Conflict and Closeness) of the Turkish and the English versions. However, item CPRS 4 was a non-significant item for Conflict in the Turkish version that affected the possibility to conduct further analyses. Findings encourage researchers to propose and assess cultural and linguistic adaptations for the Child-parent Relationship Scale before cross-cultural comparisons related to family relationships.

## Introduction

At the core of early child development is the parent-child relationship. This critical component of early experiences shapes child development and has long-term impacts throughout a person’s life span in several domains including mental health, language and cognitive skills, physical health, academic achievements, and social-emotional skills [1–3]. O’Connor and Scott [4] identified three problematic areas found in studies on parent-child relationships: (a) establishing causality to determine how parent-child relationships may influence children’s well-being and academic achievement [5, 6], (b) ensuring convertibility in order to be able to translate the results of research to family interventions [7–9]; and (c) considering the context in order to generalize findings across diverse populations and settings [10, 11]. Although the study of cultural commonalities in parent-child relationships has a long history [12], only recently scholars have examined how parents’ cultural belief and perceptions systems explain the nature and quality of parenting and family interactions in particular social contexts [13, 14]. Given the implicit nature of parents’ perceptions about their parent-child relationships in a particular cultural context [15], cross-national research entails methodological and theoretical difficulties when assessing parents’ perceptions using quantifiable measures across and within cultures [4]. Using a translated version of the Child-parent Relationship Scale (CPRS), which was originally developed based on a Western sample, this study adds to the current discussion on the cultural relevance and reliability of measures used to examine parental perceptions about parent-child relationships across socio-cultural contexts. More specifically, in assessing measurement invariance of the two constructs of the CPRS using data gathered in programs serving low-income preschool children in the U.S. and Turkey, this study aims to address current psychometric challenges identified in previous cross-national research.

While culture exerts a strong influence on family dynamics, including parenting behaviors and parent-child relationships [16–18], more studies of context are needed to be able to generalize findings across diverse populations and settings [10, 11]. Cross-cultural studies on the individualism-collectivism dichotomy [19] have identified systematic differences in parent-child relationships across cultural groups. Parents from individualistic cultures such as the U.S. tend to follow an authoritative parenting style that promotes certain child outcomes such as independence, assertiveness, and personal identity [18]. Park and colleagues [20] have argued that authoritative parenting style fosters positive parent-child relationships and limits conflict in parent-child interactions [20] . In collectivistic cultures, two cultural values–obedience and deference to parental authority–determine the nature of parent-child interaction. Yaman, Mesman, IJzendoorn, Bakermans-Kranenburg, and Linting [21] have observed that while parents from collectivistic cultures tend to adopt an authoritarian parenting style, their parenting is still characterized by warmth [17]. It is important to note that although the individualism-collectivism dichotomy is well-established in the literature, these dimensions are variably conceptualized and there is certainly diversity within each culture [22]. Turkey offers an interesting case and has been described as having characteristics of both individualistic and collectivistic cultures [23] [24]. Kağıtçıbaşı [23] has pointed out that self-reliance, warmth, affection, discipline and authoritarian control are prominent features of Turkish parenting. Dereli and Dereli [25] highlighted that child-parent relationships in Turkey differed according to family income and the educational background of parents. For instance, Saygi and Balat found that Turkish mothers with higher levels of education reported more conflictual relationships with their children [26].

In a cross-cultural study that compared Turkish and U.S samples, Aytac [27] reported that cultural differences on mothers’ values partially explained the differences in implementing positive discipline, anger, and hostility. An important limitation of this study is, however, that the comparison did not include a measurement invariance analysis of the measures. Research studies show that there are similarities across cultures, particularly in terms of how parenting and family dynamics shape children’s development [28]. Nonetheless, given that parenting beliefs and practices differ as a function of the larger cultural context [13], existing measures of parenting are limited in that they are developed mostly within Western, middle-income samples. Thus, a step in the right direction is to identify assessment tools that are validated in the cultures of interest. One way of understanding the specific pathways by which culturally-rooted practices impact child development is by employing culture-specific measures and methods of research [29]. However, using a global assessment tool is also valuable in that it allows for systematic comparison and generalization [30].

In terms of the assessment of parent-child relationships, robust measures could provide insight into the parent-child relationship and the factors affecting this relationship [31, 32]. In the US, Pianta [33] developed an instrument to measure child-parent relationships. The Child-Parent Relationship Scale (CPRS) has two forms: long (CPRS-LF, with 30 items) and short (CPRS-SF, with 15 items). Later, Driscoll and Pianta [5] conducted a study of the psychometric properties of the CPRS-SF, using correlation coefficients and internal consistency estimates. The sample included 563 children and their families (most of whom were white), who participated in the National Institutes of Child Health and Human Development Study of Early Child Care [34], a comprehensive, observational study of key developmental contexts from birth to sixth grade. Parents completed the CPRS-SF at 54 months and first grade. Participants included 294 boys and 269 girls and their fathers and mothers. Driscoll and Pianta [5] examined internal consistency across reporters and the stability of the instrument across time. Examining two subscales, Cronbach’s alphas for maternal conflict were .84 at 54 months and .84 at first grade, while Cronbach’s alphas for paternal conflict were .80 at 54 months and .78 at first grade. Cronbach’s alphas for maternal closeness were .69 at 54 months and .64 at first grade, while Cronbach’s alphas for paternal closeness were .72 at 54 months and .74 at first grade. All reported correlations were statistically significant at the .01 level. However, this validation study did not include measurement invariance analysis to examine the equivalence of the instrument with the purpose to confirm that the same constructs were measured across the reporters [5].

The factor structure of the English version of CPRS-SF was recently assessed by Dyer, Kaufman and Fagan [35], who conducted an exploratory and a confirmatory factor analysis on a sample of 420 primarily low-income fathers, mostly non-Hispanic African American. They found that the two-factor structure model had acceptable model fit, relatively high internal consistency values, and found convergent and predictive validity with various constructs measured with different instruments. Despite this sample was more ethnically diverse, the study did not test the measurement invariance in terms of race and/or ethnicity. While, according to these two papers, the two subscales of the CPRS worked well for the U.S. context in terms of internal consistency and factor structure, evidence is lacking that confirms the equivalence of the subscales across reporters with different characteristics in the American context. Potential problems may arise with regards to the validity of the evidences generated by the CPRS when it is applied in other cultures. For example, Simkiss and colleagues [36], who validated the CPRS-SF in a sample of parents of children aged 2–4 years old from the U.K, reported that item 4 *“child avoiding physical contact and affection”* neither loaded on closeness nor conflict.

In Turkey, the CPRS-LF developed by Pianta [33] was adapted into Turkish through Akgun’s dissertation work [37]. Later, Akgun and Yesilyaprak [37] conducted a study of 234 mothers of 4–6 years old children. Those authors carried out a principal component analysis (PCA) and generated two subscales, which explained 36% of the observed variance, and they dropped 6 items. The proposed two subscales were the following: Conflict (14 items) and Positive Relationship (10 items) for a total of 24 items, with alpha values of .85 and .73 for each subscale, respectively. The test-retest reliability coefficients were .98 for the Conflict subscale, .96 for the Positive Relationship subscale, and .96 for the total score [37]. In terms of Conflict, 12 items belonged to the original Conflict subscale, and 2 to the Dependency subscale. It is important to highlight that two items that were part of the original Conflict subscale -which do not appear in the final factor structure- are items 20 (“When my child is misbehaving, he/she responds to my look or tone of voice”) and 4 (“My child is uncomfortable with physical affection or touch from me”). Item 4 did not load on the subscales of this Turkish version of the CPRS-LF [37], as it was reported in UK [36].

The CPRS-LF was also adapted by Uzun and Baran [38] for a validity study of 150 preschooler’s fathers. This research reported the internal consistency and stability of the CPRS-LF over time reliability estimates. They generated 3 subscales, composed of a total of 23 items: Positive Relationship (10 items), incompatibility (7 items), and conflict (6 items). The Cronbach’s alpha reliability coefficients were .76 for the Positive Relationships subscale, .61 for Incompatibility subscale, .62 for Conflict subscale and .71 for the whole instrument [38]. Furthermore, Uzun and Baran’s work [38] included an exploratory factor analysis (EFA) of the CPRS-LF. Those authors found that 23 items loaded in three factors that explained 36.8% of the total observed variance, while the 7 other items were dropped [38].

The two studies not only revealed a factor structure of CPRS-LF that was different from the version developed by Pianta [33] for the US, but also a different factor structure between the version tested using a sample of Turkish mothers and a sample of Turkish fathers. These findings pose a challenge regarding the cross-cultural comparison of parent-child relationships within and between different groups and cultures using CPRS (e.g. male/female, immigrant/non-immigrant, ethnicity, level of education). Such comparison requires that reporters attribute the same meanings to the measured constructs of the CPRS; however, absent of measurement invariance evidence is a limitation to examine whether the different versions of CPRS measures the same constructs across diverse cultural groups and languages. Consequently, a cross-cultural discussion about cultural sensitivity, comparability and relevance of socio-emotional measures [39] like CPRS is needed to avoid misinterpretations and biased conclusions.

For this reason, to fill this gap in the literature, it is necessary to confirm the factor structure as well as to assess the equivalence of different versions of CPRS. Therefore, the purpose of the current study is to determine whether the CPRS-SF measures Conflict and Closeness in the same way in Turkey as in the U.S. If comparability is confirmed, results can be generalizable across populations and settings in the U.S. and Turkey, allowing for meaningful and valid interpretation of differences and similarities among parents [40]. In other words, this study refines the debate about the relativist cultural perspective of child-parent relationships [41] by examining whether the CPRS-SF’s subscales mean the same in different cultural contexts.

## Method

### Participants

The current study used secondary data from the Self-regulation study conducted by the University of Nebraska-Lincoln and Hacettepe University from 2014 to 2015 in the U.S and Turkey. Two convenience samples were recruited for the original study. For the Turkish sample, data were collected from low-income Turkish families with preschool children (*n* = 592) who attended public preschools in Ankara. Of parents/guardians, 32.8% reported that they had some high school education or less, while 23.3% held a high school diploma and 12.4% held a bachelor’s or higher degree. Additionally, most of the parents/guardians (73.3%) reported that they were unemployed. The U.S. sample (*n* = 4,450) consisted of English-speaking families enrolled in a high-quality preschool program serving low-income families. To qualify for the preschool program, annual household income before taxes needed to be at or below the federal poverty threshold (annual income of $23,850 for a family of four in 2014). In terms of parents/guardians´ level of education, 13.8% had some high school education or less. On the other hand, 20.5% held a high school diploma, and 14.7% held a bachelor´s or higher degree. Most of the parents/guardians (67.8%) were employed. Further characteristics of the parents/guardians’ sample are presented in Table 1.

**Table 1:** Characteristics of the sample.

Demographics	Turkish version		English version	
	*n* = 592		*n* = 4450	
	*n* (%)	Missing (%)	*n* (%)	Missing (%)
**Child's characteristics**				
**Gender**		0.0		0.0
**Male**	298 (50.3)		2138 (48.0)	
**Female**	294 (49.7)		23 l2 (52.0)	
**Parents/guardians’ employment status**		5.3		2.4
**Employed**	127 (21.5)		3017 (67.8)	
**Unemployed**	434 (73)		914 (29.5)	
**Not in labor forcé**	n.a.		410 (9.2)	
**Parents/guardians’ level of education**		25.1		1.3
**Some high school and less**	217 (36.7)		616 (13.8)	
**High school diploma**	138 (23.3)		914 (20.5)	
**Some collegue, technical training**	10 (1.7)		1774 (39.8)	
**Two year degree**	5 (0.8)		446 (10.0)	
**Bachelor degree**	59 (10.0)		423 (9.5)	
**Master degree**	7 (1.2)		166 (3.7)	
**Doctoral degree**	1(0.2)		39 (0.9)	
**Other**	6 (1.0)		15 (0.6)	

### Procedures

The study procedures were approved by the Institutional Review Boards of the University of Nebraska-Lincoln and Hacettepe University. The study was conducted in Nebraska (U.S.) and Ankara (Turkey). For the Turkish sample, data were collected by graduate students from independent preschools and kindergartens located in Altindağ, which is a socio-economically low district of Ankara. The U.S. sample included primary caregivers with children who were enrolled in Educare program, a specialized childcare program that provides education and care services for low-income families. For Turkey, data collection was carried out by graduate-level students at independent preschools and kindergartens. After obtaining a consent form from the parents/guardians for each case, the CPRS-SF and demographic questionnaires were completed by the primary caregivers. The data collectors read the questionnaires for the primary caregivers that reported difficulties to understand the questions. The number of primary caregivers who received support to complete the questionnaire in Turkey was not reported in the Self-regulation study. The data collection in the U.S. was also conducted by graduate students who provided the questionnaires to the participants. Primary caregivers also completed a questionnaire in their primary language that included demographic information and parent-child relationships data (CPRS-SF) after obtaining consent from the parents/guardians. Finally, all the de-idenfied data was provided to the researchers of the current study to conduct the secondary data analysis from the Self-regulation study.

## Measures

The instrument used in this study is the short form of the Child-Parent Relationships Scale [33], a self-report form to measure parent/guardian’s perceptions about their relationship with the child. The CPRS-SF is an adaptation of the Student Teacher Relationship Scale (STRS) [42], which assesses teachers’ perceptions of their relationships with students. The instrument is comprised of 15 items that form two subscales: Conflict and Closeness. Seven of these items load on the Closeness factor and the rest of the items load on the Conflict factor. The items are rated on a five-point Likert scale ranging from 1 (“definitely does not apply”) to 5 (“definitely applies”). The subscale score is computed as the arithmetic sum of the responded items. Turkish participants completed Turkish version of the CPRS-SF whereas American participants completed the English version. For the Turkish version, the research team took the items that comprised the short form of the CPRS from Akgun and Yesilyaprak’s work [37].

### Data analysis plan

**Fig 1:**
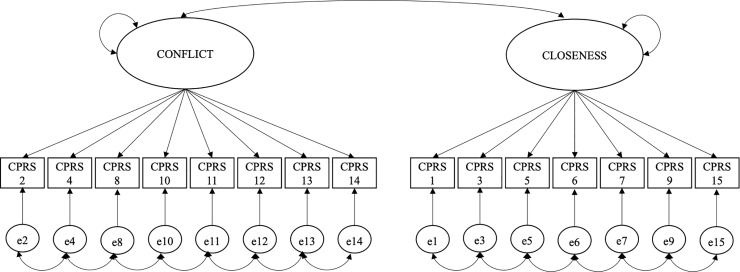
Two-factor structure for the CPRS-SF.

Single-group Confirmatory Factor Analysis (SGCFA) was first used to test factor structure for each version of the instrument as depicted in Fig 1. These two versions are considered the baseline model. No cross-factor item loading or cross-factor error correlation were allowed in the model. Later, strong measurement invariance was assessed using multigroup confirmatory factor analysis (MGCFA) in order to determine whether the original factor structure of the CPRS-SF [33] measures the same construct between the two cultural groups. Data were treated as non-normal in SGCFA, even though CFA has shown to be robust–in terms of estimates, standard errors, and fit indices–against normality violation under a number of conditions: when observed data are ordinal, with 5 or more categories, when items do not show extreme skewness and kurtotic values, when the sample is large, and when there is a relatively high number of variables per factor [43–46]. The same data treatment was maintained when doing MGCFA, considering that violation of normality assumptions in multiple-groups analysis affects goodness of fit indices and does not allow identifying the source of measurement invariance violation [47].

Following Millsap and Yung-Tein’s [48] model restriction for identification purposes, and Bowen and Masa’s [49] 4-step approach, configural invariance was first tested in order to determine whether the groups define the construct in the same way [50]. This requires that the groups exhibit the same pattern of factor loading on the subscales [51]. Second, metric invariance was evaluated to verify that the factor loadings on each subscale are the same across groups, showing equal importance to the scale. Third, scalar or between-group invariance of item thresholds was assessed (each item has *k*-1 thresholds, where *k* is the number of item-categories), while intercepts are set to zero for model identification purposes. Strong invariance is reached when configural, metric, and scalar invariance are met. That is, with strong invariance, between-group comparison of mean, variance, and covariance can be conducted at the item- and scale- level [51] and the differences can be assumed to be due to group differences in the underlying construct being measured. Fourth, strict factorial invariance or error variance equivalence was assessed to determine whether the items had the same degree of measurement error [52].

The analysis was carried out with Mplus 7.0 [53] using a robust weighted least square (WLSMV) estimator, given the non-normal distribution of the data. Therefore, the Maximum Likelihood (ML) estimator cannot be used for this type of data since normal distribution assumption of data is violated [49, 54–56]. For the same reason, a polychoric correlation matrix was used instead of the usual covariance matrix [57, 58]. Theta parameterization was employed in order to test the strict factorial invariance.

Model fit was evaluated using the Comparative Fit Index (*CFI*), the Root Mean Square Error of Approximation (*RMSEA*), and the Tucker Lewis Index (*TLI*). Model fit was considered good when .92≤*CFI*, *TLI* ≤.92 and *RMSEA*≤ .06 were observed. For the MGCFA, overall model fit was assessed for each nested model (from less to more between-group constrained). The chi-square difference (Δ*χ*^2^) test with *p* = .05 was used to evaluate the significance of changes in the model’s goodness-of-fit after every between-group constraint was added to the model. However, given that Δχ^2^ is sample dependent, Δ*CFI* ≤ -.01 was also used to assess meaningfulness of model changes [52, 59]. Partial measurement invariance is reached when the percentage of noninvariant parameters is less than 20% of all the tested parameters [51].

### Missing values

The percentage of missingness per case ranges between 0% and 100%. The majority of the participants had no missing responses (91.5%). There were only 12 cases in the Turkish sample that had missing values on all their answers; these were eliminated during the analysis. At the item level, the missingness ranges between 0.5% and 2.0%. In both samples, there were slightly more non-responses in the items related to Conflict than in the ones belonging to Closeness. No special treatment was given to the missing values since the proportion per participant was very low.

## Results

Tables 2 and 3 present the descriptive statistics at both the item and scale levels for each version of the instrument. Cronbach’s scale-level alpha shows that the Turkish version has the same degree of reliability for both scales. The English version shows different Cronbach’s alpha values for each scale, with higher values for Conflict than for Closeness. Compared to the Turkish version, the English version has a higher alpha value for Conflict and a smaller alpha coefficient for Closeness. Item average response was higher for Closeness than for Conflict in both versions of the instrument. Item-total correlation (ITC) values were above .5 for the Turkish version for all the items, except CPRS 4 (*My child is uncomfortable with physical affection or touch from me*). In the English version, this item showed a low value for Conflict. Three items belonging to Closeness in the English version showed an ITC lower than .50. The corrected ITC (CITC) showed the same pattern as the ITC. Inter-item polychoric correlation reported low coefficients for some items, and in some cases positive relationship between items of different scales (eg., CPRS 4).

**Table 2:** Descriptive statistics.

Scales	Items	Turkish							English						
		Mean	SD	Skew	Kurt	ITC	CITC	CAID	Mean	SD	Skew	Kurt	ITC	CITC	CAID
**CONFLICT**	**CPRS 2**	2.12	1.28	0.83	-0.52	0.63	0.47	0.64	1.79	1.13	1.44	1.08	0.62	0.49	0.74
	**CPRS 4**	4.25	0.97	-1.55	2.23	0.25	0.09	0.71	1.40	1.08	2.67	5.62	0.31	0.15	0.79
	**CPRS 8**	3.41	1.34	-0.42	-1.05	0.60	0.42	0.65	2.19	1.29	0.79	-0.66	0.70	0.56	0.73
	**CPRS 10**	2.36	1.38	0.60	-0.96	0.61	0.43	0.65	2.20	1.32	0.76	-0.78	0.67	0.52	0.73
	**CPRS 11**	2.79	1.40	0.21	-1.30	0.63	0.46	0.64	2.20	1.34	0.76	-0.83	0.63	0.47	0.75
	**CPRS 12**	2.82	1.47	0.18	-1.40	0.55	0.34	0.67	2.15	1.35	0.93	-0.49	0.67	0.52	0.74
	**CPRS 13**	2.63	1.35	0.27	-1.22	0.60	0.42	0.65	1.86	1.22	1.28	0.38	0.70	0.57	0.73
	**CPRS 14**	1.99	1.31	1.12	-0.03	0.55	0.37	0.66	1.91	1.27	1.15	-0.09	0.62	0.46	0.75
Cronbach's alpha	0.69								0.77						
**CLOSENESS**	**CPRS 1**	4.55	0.78	-2.44	7.06	0.58	0.41	0.66	4.95	0.30	-7.31	66.04	0.34	0.26	0.64
	**CPRS 3**	4.34	0.89	-1.71	3.10	0.56	0.35	0.67	4.81	0.62	-4.11	18.54	0.41	0.23	0.64
	**CPRS 5**	4.53	0.74	-2.03	4.91	0.62	0.46	0.65	4.89	0.49	-5.38	32.64	0.47	0.34	0.62
	**CPRS 6**	4.62	0.77	-2.63	7.66	0.64	0.49	0.64	4.88	0.49	-4.85	26.50	0.51	0.38	0.61
	**CPRS 7**	4.30	0.96	-1.60	2.38	0.62	0.40	0.66	4.22	1.25	-1.54	1.14	0.77	0.50	0.56
	**CPRS 9**	4.36	0.93	-1.83	3.43	0.54	0.32	0.68	4.62	0.78	-2.56	7.09	0.56	0.35	0.61
	**CPRS 15**	4.28	0.94	-1.56	2.35	0.62	0.42	0.66	4.37	1.16	-1.90	2.49	0.79	0.57	0.52
Cronbach's alpha	0.69								0.64						

SD: item standard deviation; Skew: skewness; Kurt: Kurtosis; ITC: item-total correlation; CITC: corrected item-total correlation; CAID: Cronbach’s alpha if item is deleted.

**Table 3:** Inter-item polychoric correlation (listwise deletion).

**Turkish (*n* = 461)**																
**Items**		**1**	**2**	**3**	**4**	**5**	**6**	**7**	**8**	**9**	**10**	**11**	**12**	**13**	**14**	**15**
**1**	**CPRS01**	1.00														
**2**	**CPRS02**	-0.33	1.00													
**3**	**CPRS03**	0.24	0.00	1.00												
**4**	**CPRS04**	0.14	0.09	0.74	1.00											
**5**	**CPRS05**	0.56	-0.30	0.43	0.34	1.00										
**6**	**CPRS06**	0.42	-0.18	0.47	0.42	0.50	1.00									
**7**	**CPRS07**	0.38	-0.11	0.22	0.23	0.40	0.48	1.00								
**8**	**CPRS08**	-0.15	0.42	0.16	0.19	-0.07	0.13	0.03	1.00							
**9**	**CPRS09**	0.21	-0.09	0.27	0.33	0.28	0.37	0.33	-0.01	1.00						
**10**	**CPRS10**	-0.25	0.46	-0.08	-0.05	-0.26	-0.13	-0.08	0.33	-0.11	1.00					
**11**	**CPRS11**	-0.15	0.32	0.04	0.09	-0.08	-0.12	-0.07	0.28	-0.12	0.27	1.00				
**12**	**CPRS12**	-0.05	0.14	0.14	0.17	-0.08	0.03	0.01	0.24	0.03	0.25	0.39	1.00			
**13**	**CPRS13**	-0.12	0.33	0.05	0.04	-0.21	-0.12	-0.04	0.32	-0.05	0.33	0.34	0.34	1.00		
**14**	**CPRS14**	-0.31	0.37	-0.09	-0.07	-0.33	-0.10	-0.12	0.28	-0.20	0.33	0.37	0.19	0.27	1.00	
**15**	**CPRS15**	0.24	-0.12	0.29	0.34	0.38	0.39	0.49	-0.02	0.35	-0.20	-0.11	-0.04	-0.10	-0.05	1.00
**English (*n* = 4154)**																
**1**	**CPRS 1**	1.00														
**2**	**CPRS 2**	-0.33	1.00													
**3**	**CPRS 3**	0.57	-0.30	1.00												
**4**	**CPRS 4**	-0.36	0.28	-0.23	1.00											
**5**	**CPRS 5**	0.65	-0.28	0.51	-0.31	1.00										
**6**	**CPRS 6**	0.51	-0.20	0.45	-0.20	0.64	1.00									
**7**	**CPRS 7**	0.21	-0.01	0.19	-0.08	0.35	0.43	1.00								
**8**	**CPRS 8**	-0.24	0.50	-0.23	0.17	-0.18	-0.07	0.03	1.00							
**9**	**CPRS 9**	0.48	-0.28	0.42	-0.17	0.50	0.49	0.33	-0.18	1.00						
**10**	**CPRS 10**	-0.17	0.39	-0.20	0.19	-0.12	-0.04	0.07	0.54	-0.18	1.00					
**11**	**CPRS 11**	-0.19	0.43	-0.14	0.19	-0.15	-0.07	-0.05	0.40	-0.18	0.36	1.00				
**12**	**CPRS 12**	-0.22	0.40	-0.18	0.19	-0.15	-0.11	-0.07	0.44	-0.21	0.48	0.46	1.00			
**13**	**CPRS 13**	-0.26	0.45	-0.28	0.29	-0.24	-0.13	-0.07	0.55	-0.26	0.48	0.44	0.54	1.00		
**14**	**CPRS 14**	-0.19	0.38	-0.13	0.17	-0.16	-0.05	0.07	0.44	-0.15	0.43	0.39	0.39	0.50	1.00	
**15**	**CPRS 15**	0.29	-0.06	0.28	-0.12	0.44	0.46	0.78	-0.04	0.41	0.04	-0.11	-0.10	-0.10	0.04	1.00

### Internal factor structure

The two-factor model presented above was evaluated for both CPRS-SF versions using single-group CFA, treating the variables as categorical. Tables 4 and 5 show the results of the model. The hypothesized model showed a close fit for the English version (*χ*^*2*^_(88)_ = 1040.049, *p* = 0.000, *CFI* = .960, *TLI* = .952, *RMSEA* = .049) after allowing error correlations between CPRS07 and CPRS15. In this version, the item CPRS 4 (*My child is uncomfortable with physical affection or touch from me*) had the lowest loading value. All the factor loadings and thresholds were statistically different from zero, and the standardized coefficients were above .45 in all cases, except that for item 4, which was .354 (*SE* = .023, *p* < .001). In the Turkish version, the model did not reach the required goodness-of-fit indices level (*χ*^*2*^_(88)_ = 1334.791, *p* = 0.000, *CFI* = .583, *TLI* = .502, *RMSEA* = .156), even after allowing within-factor error correlations. The standardized loading of the item CPRS 4 showed a negative relationship with the Conflict scale (*λ*_*4*_ = -.720, *SE* = .043, *p* < .001). This item seemed to be more related to Closeness than to Conflict, a tendency that can also be noted also in the positive correlation with the items that compose the Closeness scale (Table 2). The distribution of category responses for this item in the Turkish version was the opposite of that in the English version. That is, 96% of the participants in the Turkish sample chose “*applies somewhat*” or “*definitely applies*”, whereas 90% of the participants in the U.S. sample chose “*definitely does not apply*” or “*not really*.” Therefore, the item CPRS 4 was eliminated from the analysis in both samples, which improved the fit indices, especially in the Turkish version (*χ*^*2*^_(65)_ = 181.196, p = 0.000, *CFI* = .945, *TLI* = .924, *RMSEA* = .056). All the factor loadings again showed to be significantly different from zero in both samples. Some thresholds of 3 items of Closeness and 4 of Conflict were not significant in the Turkish sample, while they were all significant in the English version, as shown in Table 5.

**Table 4:** Fit indices for the single-group CFA.

Version	*χ*^*2*^	*Df*	*P*	*CFI*	*TLI*	*RMSEA*	*90% CI*
**Turkish**	1334.791	88	0.000	0.583	0.502	0.156	[0.149–0.164]
Turkish*	181.196	65	0.000	0.945	0.924	0.056	[0.046–0.065]
**English**	1040.049	88	0.000	0.960	0.952	0.049	[0.047–0.052]
English*	877.505	75	0.000	0.965	0.958	0.049	[0.046–0.052]

* after removing CPRS 4 from Conflict scale.

**Table 5:** Unstandardized parameter estimates and (standard errors) for SGCFA, adjusted model.

Ítems	Loadings		Thresholds							
			τ1	τ2	τ3	τ4	τ1	τ2	τ3	τ4
	Turkish	English	Turkish				English			
**CPRS01**	1.00 (0.000)	1.00 (0.000)	-3.18 (0.279)	-2.80 (0.249)	-2.42 (0.209)	-0.63 (0.102)	-4.75 (0.307)	-4.33 (0.260)	-3.69 (0.215)	-2.78 (0.168)
**CPRS02**	1.00 (0.000)	1.00 (0.000)	-0.28 (0.075)	0.62 (0.083)	1.22 (0.096)	2.01 (0.124)	0.19 (0.025)	1.19 (0.032)	1.50 (0.035)	2.30 (0.047)
**CPRS03**	0.26 (0.067)	0.73 (0.078)	-2.09 (0.124)	-1.66 (0.092)	-1.23 (0.073)	-0.09 (0.055)*	-3.04 (0.096)	-2.70 (0.083)	-2.38 (0.074)	-1.56 (0.054)
**CPRS05**	1.09 (0.238)	1.14 (0.136)	-3.68 (0.330)	-3.12 (0.275)	-2.27 (0.208)	-0.59 (0.109)	-4.27 (0.210)	-3.95 (0.195)	-3.29 (0.168)	-2.56 (0.142)
**CPRS06**	0.62 (0.119)	0.86 (0.097)	-2.60 (0.168)	-2.23 (0.141)	-1.87 (0.120)	-0.75 (0.079)	-3.69 (0.142)	-3.28 (0.123)	-2.71 (0.102)	-2.04 (0.082)
**CPRS07**	0.47 (0.088)	0.30 (0.035)	-2.16 (0.126)	-1.76 (0.101)	-1.17 (0.078)	-0.09 (0.061)*	-1.48 (0.031)	-1.24 (0.028)	-0.87 (0.024)	-0.34 (0.021)
**CPRS08**	0.58 (0.088)	1.24 (0.051)	-1.37 (0.082)	-0.65 (0.066)	-0.15 (0.062)	0.76 (0.068)	-0.33 (0.027)	0.72 (0.030)	1.11 (0.032)	2.24 (0.046)
**CPRS09**	0.67 (0.140)	0.86 (0.095)	-2.39 (0.181)	-2.01 (0.154)	-1.51 (0.122)	-0.17 (0.071)	-3.20 (0.102)	-2.65 (0.083)	-2.11 (0.068)	-0.95 (0.041)
**CPRS10**	0.83 (0.127)	1.05 (0.044)	-0.40 (0.070)	0.33 (0.071)	0.89 (0.080)	1.60 (0.099)	-0.27 (0.025)	0.64 (0.027)	0.95 (0.029)	2.04 (0.041)
**CPRS11**	0.61 (0.089)	0.89 (0.037)	-0.82 (0.068)	-0.03 (0.062)*	0.43 (0.064)	1.19 (0.075)	-0.19 (0.024)	0.58 (0.025)	0.87 (0.027)	1.87 (0.038)
**CPRS12**	0.26 (0.068)	1.10 (0.046)	-0.68 (0.060)	-0.02 (0.055)*	0.32 (0.057)	0.92 (0.065)	-0.18 (0.026)	0.76 (0.029)	1.08 (0.030)	1.82 (0.038)
**CPRS13**	0.61 (0.094)	1.38 (0.060)	-0.71 (0.066)	0.03 (0.062)*	0.54 (0.065)	1.38 (0.082)	0.27 (0.030)	1.18 (0.037)	1.55 (0.041)	2.52 (0.055)
**CPRS14**	0.75 (0.116)	0.91 (0.041)	0.06 (0.067)*	0.79 (0.076)	1.17 (0.085)	1.73 (0.103)	0.23 (0.024)	0.86 (0.027)	1.14 (0.029)	2.07 (0.041)
**CPRS15**	0.53 (0.096)	0.44 (0.048)	-2.23 (0.132)	-1.77 (0.105)	-1.23 (0.082)	0.01 (0.062)*	-1.68 (0.036)	-1.47 (0.033)	-1.14 (0.029)	-0.57 (0.024)
	**Closeness, mean**	**Conflict, mean**	**Closeness, var**	**Conflict, var**	**Correlation closeness with conflict**					
**Turkish**	0.00 (0.000)	0.00 (0.000)	1.44 (0.404)	1.00 (0.208)	-0.43 (0.092)					
**English**	0.00 (0.000)	0.00 (0.000)	1.51 (0.291)	0.72 (0.048)	-0.35 (0.044)					

Within factor error correlations are not reported

* not significant at .05.

### Measurement invariance

The MGCFA was performed on both the original and the adjusted model, which did not include the item CPRS 4. Table 6 shows the goodness-of-fit indices of the analysis. As expected, the original model did not converge in the configural analysis; thus, no further analysis was possible. Consequently, adjusted model equivalence was computed next (i.e., the factor structure without CPRS 4 and with the errors of CPRS 7 and CPRS 15 allowed to correlate). The configural invariance analysis of the adjusted model yielded close model-data fit (*χ*^*2*^_(152)_ = 1172.352, *CFI* = .961, *TLI* = .953, *RMSEA* = .052). Metric invariance and factor loading invariance results showed overall close model-data fit (*χ*^*2*^_(164)_ = 1279.120, *CFI* = .957, *TLI* = .953, *RMSEA* = .052). The chi-square difference test showed that the imposed constrains significantly worsened the model-data fit (Δ*χ*^*2*^_(12)_ = 132.982, *p* = .000), not allowing for metric invariance. The Δ*CFI*, however, yielded the opposite result (Δ*CFI<-*.01); that is, the constrains did not greatly worsen the model-data adequacy, so the analysis moved to the next step.

**Table 6:** Summary statistics of fit indexes for testing measurement invariance.

Indices	Adjusted model (after removing CPRS 04)					
	Configural	Loading	Scalar	Partial scalar	Error	Partial error
***χ***^***2***^	1172.352	1279.120	1755.004	1483.109	2099.809	1606.571
***df***	152	164	202	197	211	208
***P***	0.000	0.000	0.000	0.000	0.000	0.000
***RMSEA***	0.052	0.052	0.055	0.051	0.060	0.052
***CI*, *90%***	[0.049–0.054]	[0.049–0.055]	[0.053–0.058]	[0.049–0.053]	[0.057–0.062]	[0.049–0.054]
***CFI***	0.961	0.957	0.941	0.951	0.928	0.947
***TLI***	0.953	0.953	0.947	0.955	0.938	0.953
***Δχ***^***2***^	---	132.982	582.065	276.307	556.983	142.120
Δ***df***	---	12	38	33	14	11
Δ***p***	---	0.000	0.000	0.000	0.000	0.000
Δ***CFI***	---	-0.004	-0.020	-0.006	-0.023	-0.004

Threshold equality among samples was added to the model to test scalar invariance. As a result, the goodness-of-fit indices diminished, although they remained above the cutoff points (*χ*^*2*^_(202)_ = 1755.004, *CFI* = .941, *TLI* = .947, *RMSEA* = .055). The chi-square difference test showed that the model-data fit significantly worsened (Δ*χ*^*2*^_(38)_ = 582.065, *p* = .000) as well as Δ*CFI* (>-0.01). Therefore, partial invariance of thresholds was tested instead. The modification indices showed that the fit improved after setting thresholds for some items (i.e., items 7,8, 9 and 10) free to vary across groups. A total of 5 thresholds were unconstrained in the partial scalar model. The overall fit indices showed close fit (*χ*^*2*^_(197)_ = 1483.109, *CFI* = .951, *TLI* = .955, *RMSEA* = .051) and the chi-square difference indicated that the model significantly worsened (Δ*χ*^*2*^_(33)_ = 276.307, *p* = .000), while the Δ*CFI* again showed the opposite result (Δ*CFI<-*.01).

Residual invariance was tested next, in which all errors were set to one and the error correlation between CPRS 7 and CPRS 15 was constrained to be equal. The overall goodness-of-fit indices reported close fit, (*χ*^*2*^_(211)_ = 2099.809, *CFI* = .928, *TLI* = .938, *RMSEA* = .06), although all of them were at the limit of the cutoff point values, especially the *RMSEA*, whose upper confidence interval value was above the cutoff point. The change indices showed that the model did not fit the data in the same way as before the constraints were put in place (Δ*χ*^*2*^_(14)_ = 556.983, *p* = .000; Δ*CFI>-*.01). Item errors corresponding to CPRS 7, CPRS 8 and CPRS 15 were set to vary between groups based upon the modification indices, while the error correlation between CPRS 7 and CPRS 15 was maintained equal in both samples. This partial error invariance of the adjusted model showed good results for both overall goodness-of-fit indices (*χ*^*2*^_(208)_ = 1606.571, *CFI* = .947, *TLI* = .953, *RMSEA* = .052) and model change fit indices (Δ*χ*^*2*^_(11)_ = 142.120, *p* = .000; Δ*CFI<-*.01). Table 7 reports the unstandardized coefficients of this last model.

**Table 7:** Unstandardized MGCFA parameter estimates of the final model (partial error invariance).

**Ítems**	**Loading**	**Thresholds**			
		**τ1**	**τ2**	**τ3**	**τ4**
**CPRS01**	1.00 (0.000)	-3.02 (0.150)	-2.68 (0.125)	-2.22 (0.099)	-1.02 (0.064)
**CPRS02**	1.00 (0.000)	-0.36 (0.040)	0.61 (0.041)	0.95 (0.043)	1.73 (0.051)
**CPRS03**	0.66 (0.058)	-2.04 (0.070)	-1.68 (0.057)	-1.32 (0.049)	-0.39 (0.044)
**CPRS05**	1.15 (0.114)	-2.87 (0.134)	-2.50 (0.116)	-1.77 (0.088)	-0.77 (0.068)
**CPRS06**	0.83 (0.076)	-2.51 (0.095)	-2.09 (0.076)	-1.55 (0.062)	-0.74 (0.052)
**CPRS07**	0.68 (0.079)	-2.43 (0.124)	-1.89 (0.097)	-1.07 (0.066)	T.: -0.1 (0.063)
					E.:0.30 (0.084)
**CPRS08**	0.69 (0.057)	T.: -1.31 (0.074)	T.: -0.62 (0.061)	0.19 (0.030)	0.83 (0.063)
		E.:-0.58 (0.054)	E.:0.00 (0.028)		
**CPRS09**	0.68 (0.061)	-1.96 (0.067)	-1.46 (0.054)	-0.95 (0.047)	T.: -0.16 (0.064)
					E.:0.24 (0.052)
**CPRS10**	1.02 (0.040)	T.: -0.38 (0.066)	0.08 (0.041)	0.42 (0.041)	1.45 (0.047)
		E.:-0.86 (0.043)			
**CPRS11**	0.90 (0.035)	-0.72 (0.038)	0.05 (0.037)	0.37 (0.038)	1.34 (0.042)
**CPRS12**	1.06 (0.042)	-0.79 (0.042)	0.12 (0.041)	0.45 (0.042)	1.17 (0.045)
**CPRS13**	1.36 (0.056)	-0.56 (0.051)	0.33 (0.051)	0.75 (0.052)	1.71 (0.059)
**CPRS14**	0.87 (0.037)	-0.23 (0.037)	0.40 (0.037)	0.68 (0.038)	1.56 (0.044)
**CPRS15**	0.78 (0.086)	-2.29 (0.130)	-1.84 (0.105)	-1.18 (0.075)	0.05 (0.055)
	**Closeness, mean**	**Conflict, mean**	**Closeness, var**	**Conflict, var**	**Correlation closeness with conflict**
**Turkish**	0.000 (0.000)	0.000 (0.000)	0.85 (0.135)	0.49 (0.049)	-0.19 (0.038)
**English**	1.67 (0.145)	-0.58 (0.041)	1.90 (0.308)	0.74 (0.047)	-0.40 (0.044)

T.: Turkish version; E.: English version. Residual values are not reported.

## Discussion

This study examined the factor structure of the English and Turkish versions of the CPRS-SF in a sample of preschoolers’ parents from Turkey and the U.S. The two-factor solution showed good model fit for the English version in the sample of low-income, and educationally diverse families in the U.S. The CFA findings for the U.S. sample revealed high and statistically significant loading coefficients for all items, which is consistent with the validation study conducted by Driscoll and Pianta [5]. These authors surveyed mostly white, non-Hispanic preschoolers’ families participating in the NICHD Study of Early Child Care [34]. The findings showed incomes for these families above the federal poverty threshold ($23,850 for a family of four in 2014), while the Educare families’ annual household income before taxes were at or below the federal poverty threshold. Parents and guardians in these two samples had an average of about 15 years of education. Therefore, the findings of the present study agree with those of Driscoll and Pianta [5] in providing support for the use of the two-factor structure for the CPRS-SF English version in research carried out with English-speaking parents and guardians of similar education levels and different incomes in the American context. Although, further analysis needs to be conducted in order to verify whether this structure is maintained across different education and income levels within the U.S.

The current study showed limited support for the original factor structure of the Turkish version of the CPRS-SF. The inclusion of the item CPRS 4 (*My child is uncomfortable with physical affection or touch from me*.) in the SGCFA prevented the model from fitting the data, while it did not allow for convergence in the MGCFA. Consistently, item CPRS 4 was also found to not work well in other studies regarding CPRS-SF from U.K [36] and CPRS-LF from Turkey [37]. Unfortunately, existing results do not provide adequate theoretical or statistical explanations to interpret meaningfully the loading issues for the CPRS 4. The findings of our study could contribute to this cross-cultural discussion given the high correlation of item CPRS 4 with those included in the Closeness subscale that was found in the present study. The possible association between item CPRS 4 and closeness could be considered as an opening statement for a debate about the cultural meaning of the construct physical affection across geographical regions. Our study also provided a strong evidence that item CPRS 4 of the CPRS-SF English version worked pretty well in the Conflict scale in a U.S. sample of 4450 caregivers, which is the psychometric study with the largest sample size in the U.S. Conversely, item CPRS 4 of the CPRS-SF British English version neither loaded on closeness nor conflict in the U.K [36]. Despite these two nations are considered as individualistic cultures and their primary language is English, the factor structure is different. From this perspective, language may not directly explain the differences of factor structure between CPRS-SF in the U.S and U.K., which suggests further exploration of the particular characteristics of the reporters and their potential bias that could influence the interpretation of item CPRS 4 of the different versions of CPRS.

In line with the findings of Driscoll and Pianta [5], good model-data fit indices were observed in the original two-factor structure of the Turkish version of the CPRS-SF after removing the item CPRS 4. However, before making a decision on whether or not this item should be removed, it would be necessary to undertake a theoretical discussion of the implications of such an action on the overall structure of the CPRS. Such a discussion is needed in order to propose a culturally responsive measure to assess child-parent interaction not only in the Turkish context, but also in the global context where researchers have reported psychometric issues related to item CPRS 4 in different CPRS versions. For the purpose of this study, we specifically evaluated invariance of measurement to determine whether the items used in the adjusted model of the Turkish version of the CPRS-SF perform in the same way as those used in the English version. The first step was to establish configural invariance, which is the basis for conducting more stringent level measurement invariance tests [50]. In the measurement of the configural invariance, the constructs Conflict and Closeness were defined in the same way in both cultural contexts [50], using the adjusted model. This decision is in agreement with the work of Akgun and Yesilyaprak’s about CPRS-LF [37]. By means of a PCA, those authors also identified two factors using the long form of the CPRS. All conflict items that are in the short form of the CPRS, except CPRS 4, significantly loaded on one factor, along with other conflict items that are only present in the long form. Likewise, all the items that are in the short form of closeness significantly loaded in the second factor, along with the other items present in the long form. Since both studies reported the same findings with different samples of caregivers, we can assume that the CPRS-SF shows the same factor structure for U.S. and Turkey.

The two samples also showed metric or weak invariance (i.e., same factor loadings between groups), although only partial scalar and error invariance was attained (i.e., not the same threshold parameters across groups). This means that while factor variance and covariance comparison is possible, multi-group mean comparisons at the factor level, such as ANOVA, could not be completely free from differences in scale properties between groups. Overall, these results show that while there seems to be agreement on the existence of an underlying construct between the two groups, there is a difference in the way participants respond to the items. Studies have reported that parents socialize their children in a way that fosters values and behaviors considered adaptive to their particular culture [60]. For instance, parents from individualistic cultures like U.S. prioritize independence, assertiveness, and personal identity [18], and demonstrate sensitivity and high, firm and reasonable responsiveness toward the child [16]. On the other hand, warmth, obedience and deference to parental authority characterize the parent-child interaction in collectivistic cultures [21]. In terms of parenting style, Turkey seems to have characteristics of both individualistic and collectivistic cultures [23] [24] that may explain that constructs like parent-child conflict and parent-child closeness could be perceived similar by Turkish caregivers as well as by caregivers living in the U.S. Moreover, the present findings also revealed that caregivers from Turkey and the U.S responded differently to the CPRS items, which could impact the equivalence of CPRS-SF in Turkish and English languages. This is the first study that has examined the invariance of CPRS across individuals from different cultural contexts and provided psychometric evidence to take into consideration for conducting cross-cultural comparability of constructs like parent-child closeness and parent-child conflict in the global context. The differences among participants to respond items may be associated to cultural sensitivity issues of the instrument or particular characteristics of the reporters who participated in the study. Consequently, between-group comparison of mean, variance, and covariance at the item- and scale-level using CPRS should be performed with caution, since sample-dependence of these findings needs to be ruled out in order to avoid misinterpretation or biased conclusions.

### Limitations and future studies

The homogeneity of the sample in terms of geographical location may only reflect the conceptualization of the constructs closeness and conflict reported by low-income parents living in one city in Turkey and one state in the U.S. However, findings cannot be generalized to other cultural subgroups living in other regions of each country, which may vary according to social-cultural environments, religious and ethnic background, social values and other characteristics that may affect parent’s perceptions of the relationship with their children. In addition, results may not describe other socio-economic subgroups within countries because the current findings only deal with the understanding of parent-child interactions in low-income families. The CPRS-SF Turkish version provided some validation evidence; however, more evidence is required in order to complete the validation of the instruments among subgroups with different socioeconomic status, ethnicity, levels of education, and other key factors. Finally, the present study suggests the need for further testing of the original structure of CPRS-SF [33] in the Turkish version in order to examine the underlying relationships between the item CPRS 4 and the other items included in this version.

### Implications of the study for research

According to the findings of this study, greater recognition needs to be given to the analysis of measurement invariance of instruments that assess parenting and parent-child interactions in cross-cultural research. Researchers who aim to identify predictable and significant differences in parents’ behaviors and parent-child relationships of populations in diverse cultural environments need to perform analyses that go beyond confirmatory factor analysis, exploratory factor analysis, and validity and reliability analysis of measures. These additional types of analysis would allow researchers to ensure that the results among different cultural settings are comparable. The measurement invariance analysis reported in this paper did not show complete equivalence between the original factor structure of the English CPRS-SF version and the Turkish one. Thus, the comparison of the factor scores resulting from these versions should only be made with great caution, especially if the measure is used to assess and compare the parenting component of early childhood education programs between the U.S and Turkey. The present findings also illuminate cross-cultural studies that use any versions of CPRS-SF and CPRS-LF in the global context. To be comprehensive and culturally responsive, calculating the invariance of the CPRS might be required in order to make comparisons among countries. Additionally, the current findings reveal the importance of culture in the design and implementation of parenting programs for families with preschool children. Ultimately, the goal of cross-cultural research should be to make theory and measurement more precise by identifying the particularities of subcultural groups, even when these groups may not conform to certain theoretical assumptions that apply under different conditions (e.g., countries, ages, gender, parental cultural values, religious, etc.).

### Contributions of the study

This study has demonstrated that merely translating and adapting the CPRS to other languages does not necessarily guarantee that the resulting instrument will be sufficiently sensitive to identify the cultural and contextual variations among different countries. Consequently, it is essential to conduct measurement invariance analysis to identify the potential generalizability of the measures cross-culturally and, more specifically, to determine the comparability of the measurements obtained through the use of this standardized instrument in different language versions. This study contributes to the field of cross-cultural research by using measurement invariance to demonstrate the validity of the CPRS-SF when used to measure parents’ perceptions in two specific different cultural contexts: Turkey and the U.S. Our findings suggest that CPRS factors may fluctuate according to the culture and the linguistic background of the participants. The differences may be associated with lifestyle, parental values, historical or religious background, or other factors. This paper provides a detailed description of the process used to analyze the extent to which the CPRS-SF is able to measure similar constructs and provide comparable results. The findings of the testing suggest the need to rephrase the item CPRS 4 in the Turkish version or to consider the implications that removing this item from the scale would have for its construct validity, which is extensive for existing and future studies that report the same psychometric issues for this item in other geographical regions. Additionally, the results reveal the need to conduct further evaluations of CPRS in order to obtain exportable versions that can be used across cultures and languages. Thus, researchers would be able to compare parent-child interactions using CPRS in different cultural settings while avoiding false assumptions, attributional errors, and misleading interpretations that could undermine a solid understanding of variations and similarities of the measurements across cultures.

### Conclusion

In conclusion, the present study tested the measurement invariance of the two constructs of the CPRS-SF [33] using data gathered from programs serving low-income preschool children in the U.S. and Turkey. Since parenting and the parent-child relationship are likely to differ across cultures, to gain an accurate understanding of cultural differences, it is essential to have comparable measurement scales across cultures. This study demonstrated that, although the CPRS can be translated into other languages, further psychometric evaluations of the CPRS are needed to obtain versions of the scale that are likely to provide culturally relevant and accurate comparisons between the U.S. and Turkey, and among diverse socio-cultural groups living in different geographical regions.

## Supporting information

S1 Fig Two-factor structure for the CPRS-SF. (PDF)
