# Supplementary figures and images for: Child-parent interactions in American and Turkish families: Examining measurement invariance analysis of child-parent relationship scale
Source: PLoS One. 2020 Apr 3;15(4):e0230831. doi: 10.1371/journal.pone.0230831 (PMC7122811; doi:10.1371/journal.pone.0230831)

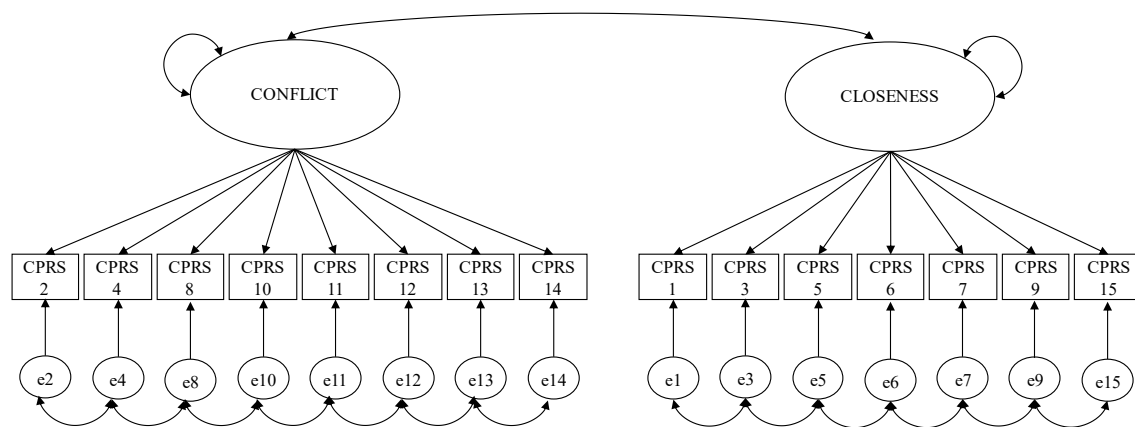

Fig 1. Two-factor structure for the CPRS-SF.

Supplement: S1 Fig — (PDF) [file pone.0230831.s001.pdf]
